# Surveillance de la paralysie flasque aiguë au Niger de 1998 à 2021

**DOI:** 10.48327/mtsi.v4i4.2024.449

**Published:** 2024-10-30

**Authors:** Maman Bachir GONI DIT ALASSAN, Zeidou Maiga ABDOULAYE, Ibrahim ALKASSOUM SALIFOU, Marie MOUNKAÏLA MIDOU, Abdoul Aziz GARBA, Mahamadou DOUTCHI, Moussa HAROUNA, Abdoul Kadir IBRAHIM MAMADOU, Eric ADEHOSSI, Joseph AKA, Saïdou MAMADOU

**Affiliations:** 1 Département de santé publique, Université André Salifou, Faculté des sciences de la santé (FSS), Hôpital national, BP 656, Zinder, Niger; 2 Département de santé publique, Université Abdou Moumouni, FSS, Hôpital national, BP 10896, Niamey, Niger; 3 Département de médecine et spécialités médicales, Université André Salifou (UAS), FSS, Hôpital national, BP 656, Zinder, Niger; 4 Hôpital de district, BP 1230, Dosso, Niger; 5 Département de médecine et spécialités médicales, Université Abdou Moumouni, FSS, Hôpital général de référence, BP 10896, Niamey, Niger; 6 Département d'informatique médicale, Université d’Abidjan, FMDS, Lomé, Cote d’Ivoire

**Keywords:** Indicateurs de performance, Paralysie flasque aigüe, Poliomyélite, Entérovirus, Surveillance épidémiologique, Niger, Afrique subsaharienne, Performance indicators, Acute flaccid paralysis, Poliomyelitis, Enterovirus, Epidemiological surveillance, Niger, Sub-Saharan Africa

## Abstract

**Introduction:**

La paralysie flasque aigüe (PFA) causée par la poliomyélite aigüe antérieure (PAA) persiste sur un mode endémique dans certains pays d’Asie et d’Afrique notamment au Niger. Des indicateurs de performance définis par l’OMS permettent d’évaluer le système de surveillance des PFA dans les pays atteints. L'objectif de cette étude est d'en mesurer les résultats au Niger de 1998 à 2021.

**Méthodologie:**

Il s'agit d'une étude transversale portant sur l'ensemble des données secondaires de la surveillance des cas de PFA notifiés au niveau de la Direction de la surveillance et de la riposte aux épidémies au Niger, sur une période de 24 ans allant de 1998 à 2021.

**Résultats:**

L’échantillon était constitué de 9 659 patients sans distinction d’âge ou de sexe. Le sex-ratio était de 1,23 et 92,01 % des patients avaient moins de 5 ans. La région de Maradi était la première des huit régions du Niger en termes de déclaration des cas (32,27 %). Elle accueille une grande partie de la population du Nigeria, pays voisin également endémique de PFA. Plus de la moitié des patients (66,59 %) avaient reçu 1 à 10 doses de vaccin antipoliomyélitique oral. De 1998 à 2021, 8 419 selles provenant de 9 494 cas (88,70 %) étaient exploitables à l'arrivée au laboratoire. Les cas de poliovirus confirmés et compatibles étaient respectivement de 0,80 % et 2,35 %. Sur les 276 patients ayant présenté une paralysie, 71,73 % souffraient d'une paralysie des deux membres, et dans 94,35 % des cas, la paralysie avait progressé en trois jours.

**Conclusion:**

Cette étude a permis d'analyser les performances du système de surveillance active de la PFA. Au Niger, cette surveillance est acceptable au regard des indicateurs de performance, mais il reste encore beaucoup d'efforts à fournir aussi bien au niveau de la population qu'au niveau du personnel de santé.

## Abstract (en)

**Introduction:**

Acute flaccid paralysis (AFP) caused by acute anterior poliomyelitis (AAP) remains endemic in some Asian and African countries, particularly Niger. Performance indicators defined by the WHO are used to evaluate the AFP surveillance system in affected countries. The objective of this study is to assess its outcomes in Niger from 1998 to 2021. The objective of this study is to assess its outcomes in Niger from 1998 to 2021.

**Methodology:**

This is a cross-sectional study based on all secondary data from AFP case surveillance reported to the Directorate of Surveillance and Epidemic Response in Niger, over a 24-year period from 1998 to 2021.

**Results:**

The sample included 9,659 patients of all ages and sexes. The sex ratio was 1.23, and 92.01% of patients were under 5 years old. The Maradi region ranked first among Niger's eight regions in terms of reported cases (32.27%). It hosts a large portion of the population from neighboring Nigeria, a country also endemic for AFP. More than half of the patients (66.59%) had received 1 to 10 doses of oral polio vaccine. From 1998 to 2021, 8,419 stool samples from 9,494 cases (88.70%) were usable upon arrival at the laboratory. Confirmed and compatible poliovirus cases accounted for 0.80% and 2.35%, respectively. Of the 276 patients who presented with paralysis, 71.73% suffered from paralysis in both limbs, and in 94.35% of the cases, the paralysis had progressed within 3 days.

**Conclusion:**

This study allowed for an analysis of the performance of the active AFP surveillance system. In Niger, this surveillance is acceptable according to performance indicators, but there is still much progress to be made at both the population level and within the healthcare workforce.

## Introduction

La paralysie flasque aigüe (PFA) de la poliomyélite aigüe antérieure (PAA) persiste sur un mode endémique dans des pays d’Asie et d’Afrique dont le Niger. La performance du système de surveillance des PFA peut être évaluée à l'aide d'une série d'indicateurs retenus par l’Organisation mondiale de la santé. La stratégie d’éradication de la PAA par l’OMS associe un programme de vaccination à l’échelle mondiale et la surveillance de tous les cas de PFA pour les enfants de 0 à 15 ans dans des pays cibles comme le Niger [12].

Le Niger a souscrit à l’éradication de la poliomyélite et réaffirmé cet engagement en 1996 à Yaoundé [6, 13]. Depuis 1997, les activités en rapport avec cet objectif (campagnes de vaccination supplémentaires, renforcement de la surveillance et de la vaccination de routine) sont organisées [15, 16]. L'augmentation de la couverture vaccinale administrative avec la 3^e^ dose de vaccin poliomyélitique oral (VPO3) en routine est passée de 62 % en 2004 à 96 % en 2012. Les campagnes supplémentaires de vaccination contre la poliomyélite ont permis d'interrompre la circulation du poliovirus sauvage (PVS) [19]. Les indicateurs de surveillance des PFA ont atteint dans toutes les régions sanitaires du pays les cibles de performance retenues. Cependant, les bons résultats enregistrés jusqu'en 2012 ont connu une baisse en 2013 avec un taux de couverture de 80 % au niveau national [13, 16]. La région de Diffa a enregistré en 2013 une couverture de 86 % en VPO3, de même que le district sanitaire de Diffa. En 2014, ce taux de couverture était de 98 % au niveau national. Trente-huit districts sanitaires ont atteint, voire dépassé, l'objectif de 80 %. Six districts (Maradi, Tchintabaraden, Bouza, Guidan Roumdji, Tchirozérine et Bilma) sont en deçà de cet objectif [9].

Depuis juin 1997, un système de surveillance des PFA a été mis en place par la Direction de la surveillance et de la riposte aux épidémies (DSRE) aux différents niveaux de la pyramide sanitaire. Le dernier cas de poliovirus sauvage type 1 (PVS1) a été relevé au Niger en 2012 [9]. La proximité avec certains pays (Nigéria et Cameroun), dans lesquels le virus de la poliomyélite a continué à circuler jusqu'en 2018, exposait le Niger à une éventuelle réintroduction du PVS [18].

Selon le rapport annuel du système national d'information sanitaire (SNIS), 25 cas de poliomyélite ont été déclarés en 2004 et 10 en 2005. Le Niger a été le premier pays en Afrique à organiser des journées nationales de vaccination (JNV) contre la poliomyélite couplées aux journées nationales des micronutriments (JNMN). Depuis 1997, il a organisé les JNV, avec des taux de couverture atteignant parfois 100 % des enfants de moins de 5 ans [16].

Malgré ces nombreuses campagnes contre la PAA, le nombre de paralysies flasques ne cesse de croître jusqu'en 2013, puis entre 2016 et 2019, posant ainsi la question de la performance des stratégies de surveillance des PFA et du rôle réel de la poliomyélite dans la survenue de ces PFA chez l'enfant. C'est dans ce cadre que nous avons réalisé ce travail dont l'objectif général est d’évaluer les indicateurs de performance des PFA au Niger de 1998 à 2021.

## Méthodologie

Le pays est subdivisé en huit régions, elles-mêmes subdivisées en départements qui, chacun, abrite un district sanitaire. L'organisation du système de santé est calquée sur le découpage administratif du pays.

**Figure 1:**
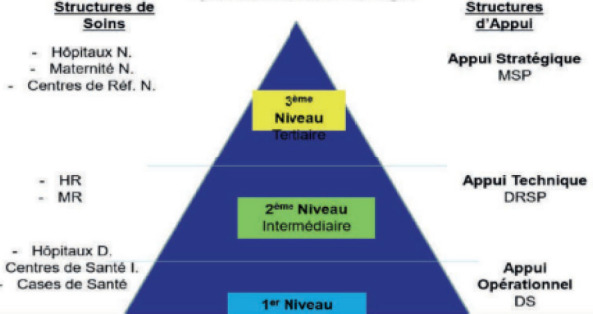
Pyramide sanitaire du Niger

La pyramide sanitaire inclut les structures publiques et privées (Fig. 1) : le niveau central est constitué d'hôpitaux, maternités et centres nationaux de référence; le niveau régional ou niveau intermédiaire est représenté par les centres hospitaliers régionaux (CHR), les centres de santé de la mère et de l'enfant (CSME); le niveau opérationnel comprend les hôpitaux de district (HD) et leurs réseaux de centres de santé [9].

Source : Kouam Camile Eric, Touré Diop Fanta, Vargas Brizuela Antonio. Sahel. Une nouvelle approche pour garantir des systèmes de santé forts et résilients. Action Contre la Faim. Juillet 2018, 20 p. DS : district sanitaire. DRSP : direction régionale de la santé publique. MSP : ministère de la santé publique. HR : hôpital régional. MR : Maternité régionale

Nous avons mené une étude transversale couvrant une période de 24 ans (1998-2021). Cette étude a porté sur l'ensemble des données secondaires de la surveillance des cas de PFA notifiés au niveau de la DSRE^1^1.La DSRE a été créée en octobre 2011 en remplacement de la direction de la statistique, de la surveillance et de la riposte aux épidémies du SNIS. La réforme de l'organisation des districts sanitaires, effective en 2014, a fait passer leur nombre de 44 à 72, nécessitant une période de rodage. au Niger pendant la période d’étude. Les cas suspects de PFA notifiés comportant au moins trois données manquantes n'ont pas été inclus. Nous avons utilisé la définition des cas suivante.

- Cas suspect : il s'agit d'une PFA chez un enfant de moins de 15 ans souffrant d'une apparition récente ou soudaine de paralysie flasque ou de faiblesse musculaire due à une cause quelconque, ou chez une personne de tout âge souffrant d'une maladie paralytique en cas de suspicion de poliomyélite par un clinicien.
- Cas probable : tout sujet de moins de 15 ans atteint de PFA ou tout sujet chez qui un médecin suspecte une poliomyélite ayant un lien épidémiologique (transmission interhumaine, antécédent de voyage dans des pays endémiques ou avec épidémies).
- Cas confirmé : tout cas probable avec au moins un des trois critères suivants : isolement dans les selles d'un PVS; isolement dans les selles d'un poliovirus dérivé d'une souche vaccinale PVDV; isolement dans les selles d'un poliovirus de type Sabin (souche vaccin oral).

Nous avons renseigné les variables suivantes.

- Variables sociodémographiques et épidémiologiques : âge, genre, provenance, statut vaccinal, semaine épidémiologique, année et date de notification.
- Variables cliniques/paracliniques : nombre de cas, contact avec un cas, douleur, fièvre, caractéristiques de la paralysie, caractéristiques des selles.

Les indicateurs de performance ont été les suivants :

- Indicateur 1 = taux de PFA non poliomyélitique chez les enfants de moins de 15 ans. C'est le nombre de cas de PFA non poliomyélitique enregistrés pour 100 000 enfants de moins de 15 ans (objectif OMS > 2).
- Indicateur 2 = pourcentage des cas de PFA pour lesquels 2 prélèvements sont recueillis dans les 14 jours (objectif OMS > 80 %).
- Indicateur 3 = pourcentage de cas de PFA pour lesquels un suivi à 60 jours est effectué pour vérifier si le patient présente une paralysie résiduelle (objectif OMS > 80 %).
- Indicateur 4 = pourcentage des prélèvements arrivés au laboratoire moins de 3 jours après leur envoi (objectif OMS > 80 %).
- Indicateur 5 = pourcentage des prélèvements de selles arrivés au laboratoire dans de bonnes conditions : température > 8°C, volume des selles, absence de dessiccation (objectif OMS > 80 %).
- Indicateur 6 = pourcentage des prélèvements pour lesquels les résultats sont envoyés dans les 28 jours après leur réception au laboratoire (objectif OMS > 80 %).
- Indicateur 7 = pourcentage des prélèvements pour lesquels un entérovirus non-poliovirus est isolé (objectif OMS > 10 %).
- Indicateur 8 = pourcentage des cas investigués dans les 48 heures après notification (objectif OMS > 80 %).

Une fiche d'investigation (Annexe 1) a permis de recueillir les informations relatives à l'identification du sujet, aux données sociodémographiques, manifestations cliniques, historique de la maladie (date de début de la paralysie, présence de fièvre, progression de la paralysie dans les 4 jours, asymétrie de la paralysie), statut vaccinal et moment de prélèvement d’échantillons, suivi après 60 jours pour les cas inadéquats (cas prélevés après 14 jours suivant le début de la paralysie, cas avec intervalle de prélèvement des deux échantillons supérieur à 48 h, quantité insuffisante de selles, échantillon arrivé en mauvaises conditions au laboratoire).

L'analyse de la performance de la surveillance des PFA a été faite à l'aide des indicateurs standards de l’OMS.

Une revue documentaire de toutes les données des maladies à déclaration obligatoire (MDO) de la DSRE, complétées avec les listes linéaires de l’OMS, a été faite. Ces données ont été collectées à l'aide de la fiche d'investigation (Annexe 1). Les données ont été traitées et analysées à l'aide des logiciels Excel 2013 et Epi Info™ dans sa version 7.2.2.6. Les tableaux et les représentations graphiques ont été réalisés à l'aide du logiciel Excel. La méthode de double saisie nous a aidés à concevoir la base des données. Le nettoyage de la base a permis de supprimer les doublons. Les données manquantes ont été supprimées de la base des données. Les variables quantitatives ont été présentées sous forme des moyennes et de leur écart-type ou leur intervalle de confiance IC à 95 %, les variables qualitatives sous forme d'effectifs et de pourcentages. Les proportions ont été comparées à l'aide du test statistique du Chi-deux de Pearson au seuil de 5 %. Tous les indicateurs ont été calculés et comparés aux normes standards de l’OMS.

Nous avons bénéficié d'une autorisation de recherche de la Faculté des sciences de la santé (FSS) de l’Université Abdou Moumouni (UAM) de Niamey et de l'approbation des autorités de la DSRE. L'anonymat et la confidentialité des données ont été assurés par la suppression des données identifiantes. Les données ont été sécurisées et n'ont servi qu’à cette étude. Elles seront détruites après une durée minimum de trois ans.

## Résultats

Pendant ces 24 années, un échantillon de 9 659 patients a été obtenu à travers le système national de surveillance. Cela constitue l'ensemble des données fournies par le système de surveillance épidémiologique des huit régions du Niger à travers les MDO centralisées au niveau de la DSRE.

**Figure 2:**
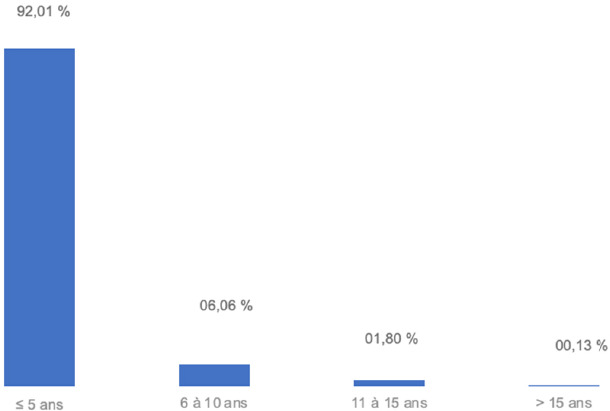
Répartition de l’échantillon des cas de PFA selon la tranche d’âge au Niger de 1998-2021

Le sex-ratio était de 1,23 (sexe masculin : 55,2 %). La Figure 2 nous donne le nombre des cas selon la tranche d’âge et le Tableau I nous montre la provenance des cas. L’âge moyen était 4,09 ans (ET = 1,2 ans) et la tranche d’âge de 0 à 5 ans dominait (92 %). Les cas provenaient majoritairement de la région de Maradi.

**Tableau I:** Répartition de l’échantillon selon la région de provenance des cas de PFA au Niger de 1998-2021

Régions	Effectifs	%
Agadez	192	1,99
Diffa	1 303	13,49
Dosso	682	7,06
Maradi	3 117	32,27
Niamey	329	3,41
Tahoua	1 028	10,64
Tillabéry	952	9,86
Zinder	2 056	21,29
Total	9 659	100

**Figure 3:**
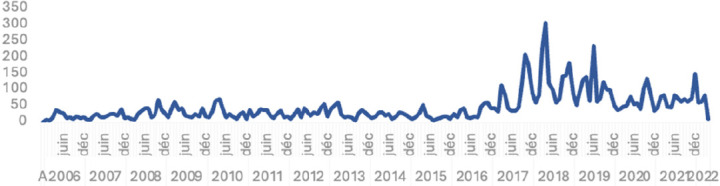
Évolution des cas suspects de PFA au Niger de 1998-2021

L’évolution des cas des PFA au Niger de 1998 à 2021 est résumée dans la Figure 3. Le nombre annuel de cas de PFA est resté constant de 2006 à 2016, puis a été irrégulier avec des pics récurrents jusqu'en 2022.

Les antécédents et signes cliniques des patients sont présentés dans les Tableaux II et III.

**Tableau II:** Répartition des patients selon le contact avec un cas suspect de PFA, l'apparition du symptôme au début de la maladie et leur hospitalisation sur la période 1998-2021

Variables	Effectifs	Pourcentage (%)
**Contact avec un cas**		
oui	4 432	65,09
non	2 377	34,91
**Total**	**6 809**	**100**
**Douleur**
oui	1 777	61,04
non	1 134	38,96
**Total**	**2 911**	**100**
**Fièvre**		
oui	6 857	96,07
non	280	3,93
**Total**	**7 137**	**100**
**Hospitalisation**		
oui	953	12,99
non	6 383	87,01
**Total**	**7 336**	**100**

**Tableau III:** Répartition des patients selon le mode d'installation, la localisation, l'asymétrie, la progression de la paralysie et les doses de VPO reçues au Niger de 1998 à 2021

Variables	Effectifs	Pourcentage (%)
**Installation soudaine de la paralysie**		
oui	7 031	98,97
non	73	1,03
**Total**	**7 104**	**100**
**Localisation de la paralysie (membre)**		
1	64	23,18
2	198	71,73
3	68	2,17
4	8	2,92
**Total**	**276**	**100**
**Asymétrie d'un membre inférieur**		
oui	2 383	94,35
non	4 316	5,65
**Total**	**6 699**	**100**
**Nombre de doses de VPO reçues**		
0	165	1,72
1-2	2 203	23,05
3-10	4 161	43,54
> 10	3 027	31,69
**Total**	9 556	100

Les patients ayant fait l'objet d'une injection avant la survenue de la maladie représentaient 10,32 % des effectifs.

Les données paracliniques sont décrites dans le Tableau IV.

**Tableau IV:** Répartition des patients en fonction des caractéristiques des selles prélevées (1998-2021)

Variables	Effectifs	Pourcentage (%)
**État des selles**		
adéquat	8 419	88,7
adéquat	1 073	11,3
**Total**	**9 492**	**100**
**Culture finale**		
négatif	6 587	70,26
suspect poliovirus	866	9,24
suspect poliovirus + entérovirus non-polio	36	0,38
entérovirus non-polio	1 886	20,12
**Total**	9 375	**100**
**Classification finale des cas**		
polio confirmée	58	0,80
compatibles	169	2,35
non inclus	6 960	96,85
**Total**	**7 187**	**100**

Les différents délais de notification, d'investigation et d'admission des cas sont résumés dans les Tableaux V et VI.

**Tableau V:** Répartition des patients selon le temps entre la survenue et la notification de la maladie, la notification et l'investigation des cas, et entre la survenue de la maladie et l'admission des cas au Niger (1998-2021)

Variables	Effectifs	Pourcentage (%)
**Délai entre la survenue de la maladie et la notification (en jours)**		
<1	374	5,2
2-3	1 209	16,83
>3	5 598	77,97
**Total**	**7 181**	**100**
**Délai entre la notification et l'investigation des cas**		
≤ 48 heures	9 457	98,07
> 48 heures	186	1,93
**Total**	9 643	**100**
**Délai entre la survenue de la maladie et l'admission (en jours)**		
1	84	11,18
2-3	154	20,5
>3	513	68,32
**Total**	**751**	**100**

**Tableau VI:** Répartition des patients selon le temps mis entre les deux prélèvements, l'envoi et la réception du prélèvement au laboratoire et entre la survenue de la maladie et le premier prélèvement (1998-2021)

Variables	Effectifs	Pourcentage (%)
**Délai entre le premier et le deuxième prélèvement**		
≤ 48 heures	7 248	99,72
> 48 heures	20	0,28
**Total**	**7 268**	**100**
**Délai entre le prélèvement et l'envoi au laboratoire**		
≤ 72 heures	477	6,82
> 72 heures	6 517	93,18
**Total**	**6 994**	**100**
**Délai entre l'envoi et la réception au laboratoire**		
≤ 72 heures	7 124	77,66
> 72 heures	2 049	22,34
**Total**	**9 173**	**100**
**Délai entre la survenue de la maladie et le premier prélèvement (en semaines)**		
2	6 326	87,37
4	570	7,87
6	238	3,28
> 6	106	1,48
**Total**	**7 240**	**100**

Dans 22 % des cas, la notification a été faite dans un délai inférieur ou égal à trois jours après la survenue de la maladie. L'investigation a réalisée en retard avec un délai qui va au-delà de 48 heures pour 2 % des cas. Dans 34 % des cas, le délai entre la survenue de la maladie et l'admission était inférieur ou égal à trois jours.

Le Tableau VII précise les différents niveaux atteints par les indicateurs en fonction des seuils définis par l’OMS.

**Tableau VII:** Répartition des indicateurs du système de surveillance de la PFA au Niger, 1998-2021

Indicateur	Norme OMS	Résultats Niger	Commentaire	Comment
Taux de PFA non polio chez > 15 ans	> 2	1,5	Beaucoup de districts étaient silencieux, ne notifiaient pas ou notifiaient moins, surtout au moment de la réorganisation du système sanitaire du Niger à partir de 2014 (passage de 44 à 72 districts).	Many districts were silent, did not report, or reported less, especially at the time of the reorganization of the health system in Niger from 2014 (increase from 44 to 72 districts).
Pourcentage de cas de PFA pour lesquels 2 prélèvements sont recueillis dans les 14 jours	≥ 80 %	100 %	L'objectif est atteint du fait que l'investigation se fait en collaboration avec les agents du district et les partenaires en plus des agents du centre de santé intégré.	The objective was achieved because the survey was carried out in collaboration with district agents and partners, in addition to the agents of the integrated health center.
Pourcentage de cas de PFA pour lesquels un suivi à 60 jours est effectué	≥ 80 %	92 %	Objectif atteint, voire dépassé, grâce à l'implication des acteurs.	The goal was achieved and exceeded thanks to the involvement of all stakeholders.
Pourcentage de prélèvements de selles arrivés au laboratoire dans de bonnes conditions	≥ 80 %	89 %	La formation des agents et la mise en place d'un système de surveillance cas par cas pour la poliomyélite au niveau national à travers la DSRE.	Training of agents and implementation of a case-based surveillance system for poliomyelitis at the national level through the DSRE.
Pourcentage de prélèvements pour lesquels les résultats sont envoyés dans les 28 jours après leur réception au laboratoire	≥ 80 %	83 %	Un mécanisme de transport était mis en place avec la sous-traitance des compagnies de transport.	A transport mechanism was set up by subcontracting transport companies.
Pourcentage de prélèvements pour lesquels un entérovirus non-poliovirus est isolé	> 10 %	30 %	Le renforcement de capacité des laboratoires et leur implication dans le système de surveillance surtout avec des modules spécifiques dans le document du système intégré des maladies et riposte (SIMR) plus précisément la SIMR 4.	Capacity building for laboratories and their involvement in the surveillance system, especially with specific modules in the Integrated Disease and Response System (IDRS) document, particularly IDRS 4.
Pourcentage de cas investigués dans les 48 heures après notification	≥ 80 %	98 %	L'objectif est aussi atteint du fait de la mise en place d'un système de surveillance à base communautaire avec l'implication des relais communautaires. Plusieurs stratégies déterminantes, dont celle de l’OMS dénommée autovisual AFP detection and reporting (AVADAR), qui permet de déclencher une alerte au niveau communautaire. Cette alerte passe par le centre de santé intégré, le district sanitaire, le DRSP, la DSRE, l’OMS pays et jusqu'au niveau OMS sous régional et dès là un rapport d'investigation est attendu au niveau périphérique dans les 24 à 48 heures qui suivent.	This goal has also been achieved through the establishment of a community-based surveillance system involving community relays. There are several key strategies, including the WHO's Automatic Visual AFP Detection and Reporting (AVADAR) strategy, which triggers an alert at the community level. This alert goes through the integrated health center, the health district, the DRSP, the DSRE, the WHO country, and up to the WHO subregional level. An investigation report is then expected at peripheral level within 24 to 48 hours.

## Discussion

Plusieurs indicateurs ont atteint les seuils de l’OMS et certains ont même dépassé ceux-ci. Cela résulte de plusieurs efforts et stratégies mis en œuvre, passant de la riposte vaccinale au système de surveillance communautaire mis en place au niveau du pays avec le soutien des partenaires dont l’OMS. Le seul indicateur resté en dessous du seuil OMS est le taux de PFA non polio chez les enfants de moins de 15 ans (1,5 au lieu de > 2). Le système sanitaire du Niger a connu à partir de 2014 une réorganisation avec la création de plusieurs districts à partir des districts existants, leur nombre passant de 44 à 72 avec une période d'adaptation variable. Les ressources et infrastructures (notamment les chaînes de froid, les réactifs et consommables de la vaccination) n'ont pas toujours suivi. Seuls les plans d'action annuels des anciens districts sanitaires ont été élaborés, validés et financés. Les activités des nouveaux districts découlent de la mise en œuvre stricte de celles des anciens où ils sont toujours considérés comme des centres de santé intégrés des districts mères. Les indicateurs de la surveillance sont attendus des nouveaux districts au même titre que des anciens. Par manque des moyens, les nouveaux districts n'ont pas notifié les cas suspects de PFA. La non prise en compte de toutes les données explique en grande partie les différences d'effectifs entre les indicateurs. La notification des cas a été réalisée au niveau communautaire par des télégrammes hebdomadaires concernant les maladies à déclaration obligatoire sans que les investigations ne soient menées systématiquement. Ces lacunes ont perduré plusieurs années au début de la surveillance.

Cette étude ainsi que plusieurs autres, montre un sex-ratio en faveur du genre masculin [1, 6, 7, 18]. Les garçons, plus souvent livrés à eux-mêmes dans la petite enfance que les fillettes, entrent plus précocement en contact avec les sources d'infections (virales, parasitaires, bactériennes…) à travers les jeux et les expositions à l'environnement insalubre.

La tranche d’âge des moins de cinq ans est plus fréquemment concernée par la PFA [2, 4, 10]. Les virus sont largement présents dans l'environnement. D'autres causes, comme les multiples injections intramusculaires faites notamment lors de traitements antiinfectieux, peuvent être à l'origine de séquelles paralysantes. Par ailleurs, les agents de santé recherchent et rapportent davantage les cas suspects de PFA chez les enfants de moins de cinq ans que chez les plus âgés. Les moins de cinq ans sont plus facilement accessibles du fait qu'ils sont concernés par la plupart des programmes verticaux de vaccination, de distribution des médicaments et de lutte contre la malnutrition à travers les différents centres de récupération nutritionnelle.

Chez la plupart des patients, la PFA est associée à de la fièvre, ce qui permet de la distinguer d'un syndrome de Guillain Barré. La fièvre est présente avant, pendant et après l'installation de la paralysie dans la névrite. Elle est rare dans la myélite transverse. La fièvre peut aussi être due à l'association d'une ou de plusieurs autres maladies infectieuses. Lorsqu'elle est associée à une paralysie flasque d'installation rapide en 72 heures, disparaissant peu après l'installation de la paralysie, elle évoque une poliomyélite [8]. Dans cette série, près d'un tiers des PFA présente une paralysie asymétrique des membres, touchant le plus souvent les membres inférieurs, les muscles proximaux (quadriceps, deltoïde), de façon relativement anarchique, ce qui est caractéristique de la poliomyélite [3, 5, 8]. L'intensification des campagnes de vaccination au cours de ces dernières années au Niger pourrait expliquer la baisse du nombre de cas de poliomyélite. En outre, la surveillance est renforcée grâce à de nouvelles stratégies telles que *l’Auto-Visual AFP Detection and Reporting* (AVADAR). Il s'agit d'une application logicielle mobile basée sur les SMS, conçue pour améliorer la qualité et la sensibilité de la surveillance de la PFA par les agents de santé et les informateurs clés au sein des établissements hospitaliers et des communautés locales. Enfin, la surveillance environnementale a été améliorée au Niger depuis 2014 à travers des sites sentinelles puis mis à l’échelle entre 2015 et 2018.

La qualité et la rapidité d'expédition des selles – idéalement moins de 72 heures après leur émission – est une condition essentielle de succès du diagnostic [18, 19]. Il n'est pas toujours possible d'imputer certains cas de PFA à des entérovirus non-polio. La fréquence des poliovirus sauvages et vaccinaux ainsi que des entérovirus non-polio, particulièrement parmi les enfants sans symptômes, varie selon les conditions de diagnostic et les pays [2, 18]. La réversion vers la neurovirulence du poliovirus vaccinal pourrait s'expliquer par une variabilité génétique au cours de laquelle ces souches acquièrent dans l'intestin un phénotype partiellement neurovirulent. Ces paralysies post vaccinales doivent être systématiquement recherchées aussi longtemps que dure l'utilisation du vaccin poliomyélitique oral. Dans certains pays tels que la France, le vaccin injectable est préféré à cause de ces réversions [12, 14, 17].

Selon l’OMS, au moins 80 % des cas de paralysies flasques aiguës doivent être accompagnés de deux échantillons de selles prélevées entre 24 à 48 heures d'intervalle et dans les deux semaines suivantes [14]. Un système de surveillance est considéré comme performant lorsque 80 % des échantillons sont prélevés dans les 14 jours [14]. Au Niger, les selles sont analysées au laboratoire de référence de l’Institut Pasteur de Dakar. Le dernier cas de poliomyélite à virus sauvage a été rapporté en 2012 dans le district sanitaire de Keita (région de Tahoua), et le pays a été déclaré libre de poliovirus sauvage en juillet 2016 par la Commission régionale de certification pour l’éradication de la poliomyélite en Afrique. Cependant, depuis septembre 2020, des cas de poliovirus dérivés de souche vaccinale de type 2 sont observés, avec neuf cas importés du Nigéria, du Togo et de la Côte d’Ivoire [13, 16]. À partir de 2020, l'implication des personnels de santé dans la gestion de la pandémie de COVID-19 et la fermeture des voies aériennes et terrestres ont eu un impact négatif sur la surveillance des PFA. Cela s'est traduit par la baisse du nombre de cas notifiés, la qualité médiocre des échantillons et les retards dans leur acheminement dans les laboratoires régionaux de référence : 365 échantillons sont ainsi restés stockés au niveau central pendant plusieurs mois. Il est donc nécessaire de doter le pays d'un laboratoire spécialisé permettant d'accroître la rapidité de la détection des poliovirus pour la différenciation entre poliovirus sauvage et poliovirus dérivé de la souche vaccinale. Il faut maintenir la surveillance en insistant sur les tâches suivantes : a) prélever systématiquement tous les cas suspects des cas de PFA; b) acheminer au laboratoire de référence les selles suspectes en respectant les conditions de prélèvement, de conservation et d'acheminement; c) investiguer systématiquement tous les cas de PFA et d) engager les mesures de riposte.

Depuis 1998, on peut dire que le système de surveillance du Niger est performant car la plupart des indicateurs est supérieure ou égale à 80 % conformément aux recommandations de l’OMS (Tableau VII). Dans cette série étude, la prévalence la plus élevée des cas de poliomyélite a été observée dans la région de Maradi. L'année 2018 a présenté le plus grand nombre de cas. Le statut vaccinal des enfants montre un niveau d'immunité acceptable contre la poliomyélite. La progression de la maladie sur trois jours a été davantage observée chez les patients non vaccinés. Les nouvelles recommandations émises par l’OMS au sujet des vaccins et de la surveillance de la PFA sont essentielles à l’éradication de la poliomyélite. Au Niger, la surveillance épidémiologique et en laboratoire de façon continue est importante afin de maintenir la certification de pays exempt de poliomyélite à un moment crucial de l’éradication de cette maladie dans le monde. Toutefois, ce résultat ne pourra être obtenu qu'en faisant preuve d'une vigilance clinique continue en ce qui concerne la PFA, laquelle devra comprendre des protocoles appropriés en matière de déclaration et d'analyses.

## Conclusion

Au cours de ces 24 dernières années, la surveillance active des PFA au Niger au vu des indicateurs de performance est globalement acceptable, bien qu'il y ait encore des efforts à fournir aussi bien au niveau de la population (respect des mesures d'hygiène alimentaire et environnementale), qu'au niveau des services de santé (implication nécessaire de la communauté dans toutes les activités de surveillance à travers l’éducation et l'information). La surveillance épidémiologique est importante afin de maintenir la certification de pays exempts de poliomyélite. Ce résultat pourra être obtenu en faisant preuve d'une vigilance continue concernant la PFA.

## Financement de l’étude

L’étude n'a bénéficié d'aucun financement.

## Contribution des auteurs

ABDOULAYE Z et ALKASSOUM S.I : épidémiologistes et collaborateurs du département santé publique de l’Université Abdou Moumouni, impliqués dans la recherche depuis le protocole jusqu’à l'analyse des données

MARIE M.M : Étudiante en 8ème année d’études médicales, impliquée dans l'extraction des données

GARBA AA, HAROUNA M, ABDOL KADIR I.M, MAHAMANE D.M : internistes et infectiologues dans les régions de Dosso, Maradi, Zinder et de l’UAS, impliqués dans la collecte et l'analyse des données

ADEHOSSI E.O : Professeur titulaire en médecine interne, impliqué dans l'accès à certaines sources d'information et la rédaction de l'article

AKA J : Professeur agrégé en épidémiologie, pour appréciation de tout le travail

SAIDOU M : Professeur en bactériologie – virologie, pour la validation de tout le travail.

## Conflit d'intérêt

Les auteurs ne rapportent aucun conflit d'intérêt.
