# Supplementary material for: Acute flaccid paralysis surveillance in Niger from 1998 to 2021
Source: Med Trop Sante Int. 2024 Oct 30;4(4):mtsi.v4i4.2024.449. [Article in French] doi: 10.48327/mtsi.v4i4.2024.449 (PMC11892389; doi:10.48327/mtsi.v4i4.2024.449)
Supplement: Related texts

# Research article: Acute flaccid paralysis surveillance in Niger from 1998 to 2021

## Introduction

Acute flaccid paralysis (AFP) due to acute anterior poliomyelitis (AAP) is endemic in several Asian and African countries, including Niger. The performance of the AFP surveillance system can be assessed using a set of indicators defined by the World Health Organization. The WHO's AFP eradication strategy combines a global immunization program with surveillance of all AFP cases in children aged 0-15 years in target countries such as Niger [12].

Niger committed itself to the eradication of poliomyelitis and reaffirmed this commitment in 1996 in Yaoundé [6, 13]. Since 1997, activities related to this objective (supplementary immunization campaigns, strengthening of surveillance and routine immunization) have been organized [15, 16]. The increase in administrative immunization coverage with the third dose of routine oral polio vaccine (OPV3) has risen from 62% in 2004 to 96% in 2012. Additional polio immunization campaigns have interrupted the circulation of wild poliovirus (WPV) [19]. AFP surveillance indicators in all health regions of the country have met performance targets. However, the good results recorded until 2012 were followed by a decline in 2013, with a national coverage rate of 80% [13, 16]. The Diffa region recorded 86% OPV3 coverage in 2013, as did the Diffa health district. In 2014, the national coverage rate was 98%. Thirty-eight health districts met or exceeded the 80% target. Six districts (Maradi, Tchintabaraden, Bouza, Guidan Roumdji, Tchirozérine and Bilma) are below this target [9].

Since June 1997, the Direction de la surveillance et de la riposte aux épidémies (DSRE) has established an AFP surveillance system at the different levels of the health pyramid. The last case of wild poliovirus type 1 (WPV1) was reported in Niger in 2012 [9]. Niger's proximity to certain countries (Nigeria and Cameroon), where poliovirus continued to circulate until 2018, exposed it to a possible reintroduction of WPV [18].

According to the annual report of the National Health Information System (SNIS), 25 cases of poliomyelitis were reported in 2004 and 10 in 2005. Niger was the first country in Africa to organize National Immunization Days (JNV) against poliomyelitis, coupled with National Micronutrient Days (JNMN). It has organized NIDs since 1997, with coverage rates sometimes reaching 100% of children under 5 years of age [16].

Despite these numerous campaigns against AFP, the number of cases of flaccid paralysis continued to increase until 2013 and again between 2016 and 2019, raising questions about the effectiveness of AFP surveillance strategies and the actual role of poliomyelitis in the occurrence of AFP in children. It is in this context that we have carried out this work, the general objective of which is to evaluate AFP performance indicators in Niger from 1998 to 2021.

## Methodology

The country is divided into eight regions, which in turn are divided into departments, each of which has a health district. The organization of the health system is based on the administrative division of the country.

The health pyramid includes public and private structures (Fig. 1): the central level is composed of hospitals, maternity wards and national reference centers; the regional or intermediate level is represented by regional hospital centers (CHR) and mother and child health centers (CSME); the operational level includes district hospitals (HD) and their networks of health centers [9].

**Figure 1:** Niger health pyramid

Source : Kouam Camile Eric, Touré Diop Fanta, Vargas Brizuela Antonio. Sahel. Une nouvelle approche pour garantir des systèmes de santé forts et résilients. Action Contre la Faim. Juillet 2018, 20 p. DS : district sanitaire. DRSP : direction régionale de la santé publique. MSP : ministère de la santé publique. HR : hôpital régional. MR : Maternité régionale

We conducted a cross-sectional study over a 24-year period (1998-2021). This study included all secondary surveillance data on AFP cases reported to the DSRE^1^1.The DSRE was established in October 2011, replacing the Directorate of Statistics, Surveillance, and Epidemic Response of the SNIS. The reorganization of health districts, which took effect in 2014, increased their number from 44 to 72, requiring a transition period. in Niger during the study period. Suspected AFP cases reported with at least three missing data were not included.

The following case definition was used.

- Suspected case: AFP in a child under 15 years of age with recent or sudden onset of flaccid paralysis or muscle weakness from any cause, or in a person of any age with a paralytic illness if poliomyelitis is suspected by a physician.
- Probable case: any person under 15 years of age with AFP, or any person in whom a physician suspects poliomyelitis with an epidemiologic link (person-to-person transmission, history of travel to endemic or endemic countries).
- Confirmed case: any probable case with at least one of the following three criteria: stool isolation of PVS; stool isolation of poliovirus derived from a PVDV vaccine strain; stool isolation of Sabin-type poliovirus (oral vaccine strain).

The following variables were collected.

- Sociodemographic and epidemiologic variables: age, gender, origin, vaccination status, epidemiologic week, year and date of notification.
- Clinical/paraclinical variables: number of cases, case contact, pain, fever, paralysis characteristics, stool characteristics.

The performance indicators were as follows:

- Indicator 1 = Non-polio AFP rate in children under 15. This is the number of non-polio AFP cases detected per 100,000 children under 15 (WHO target > 2).
- Indicator 2 = percentage of AFP cases for which 2 samples are collected within 14 days (WHO target > 80%).
- Indicator 3 = percentage of AFP cases followed up for 60 days for residual paralysis (WHO target > 80%).
- Indicator 4 = percentage of specimens arriving at the laboratory less than 3 days after shipment (WHO target > 80%).
- Indicator 5 = percentage of stool samples arriving at the laboratory in good condition: temperature > 8°C, stool volume, absence of desiccation (WHO target > 80%).
- Indicator 6 = Percentage of samples for which results are sent within 28 days of receipt at the laboratory (WHO target > 80%).

Indicator 7 = Percentage of specimens in which a non-poliovirus enterovirus is isolated (WHO target > 10%).

Indicator 8 = percentage of cases investigated within 48 hours of notification (WHO target > 80%).

An investigation form (Appendix 1) was used to collect information on case identification, sociodemographic data, clinical manifestations, disease history (date of onset of paralysis, presence of fever, progression of paralysis within 4 days, asymmetry of paralysis), immunization status and time of sample collection, 60-day follow-up of inadequate cases (cases collected after 14 days from onset of paralysis, cases with two sample collection intervals greater than 48 hours, inadequate stool quantity, sample arrived at the laboratory in poor condition).

The analysis of AFP surveillance performance was based on standard WHO indicators.

A literature review of all notifiable disease data from the DSRE, supplemented by WHO linear lists, was conducted. These data were collected using the survey form (Appendix 1).

The data were processed and analyzed using Excel 2013 and Epi Info™ software version 7.2.2.6. Tables and graphs were created using Excel. The double-entry method was used to construct the database. The database was cleaned to eliminate duplicates. Missing data was removed from the database. Quantitative variables were presented as means and their standard deviation or 95% CI confidence interval, and qualitative variables were presented as numbers and percentages. Proportions were compared using Pearson's chisquare statistical test at the 5% threshold. All indicators were calculated and compared with standard WHO norms.

We had a research authorization from the Faculty of Health Sciences (FSS) of the Abdou Moumouni University (UAM) of Niamey and approval from the DSRE authorities. Anonymity and confidentiality of the data were ensured by removing identifying information. The data were secured and used only for this study. They will be destroyed after a minimum of three years.

## Results

Over the past 24 years, a sample of 9,659 patients was obtained from the national surveillance system. This represents all the data provided by the epidemiological surveillance system in the eight regions of Niger through the MDOs centralized at the DSRE.

The sex-ratio was 1.23 (male: 55.2%). Figure 2 shows the number of cases by age group, and Table I shows the origin of the cases.

**Figure 2:** Distribution of the AFP case sample by region of origin in Niger, 1998-2021

**Table I:** Distribution of the sample by region of origin of AFP cases in Niger from 1998-2021

Regions	Sample size	%
Agadez	192	1,99
Diffa	1 303	13,49
Dosso	682	7,06
Maradi	3 117	32,27
Niamey	329	3,41
Tahoua	1 028	10,64
Tillabéry	952	9,86
Zinder	2 056	21,29
Total	9 659	100

The mean age was 4.09 years (SD = 1.2 years), with the 0-5 age group dominating (92%).

The majority of cases were from the Maradi region.

The evolution of AFP cases in Niger from 1998 to 2021 is summarized in Figure 3. The annual number of AFP cases remained constant from 2006 to 2016, then was irregular with recurrent peaks until 2022.

**Figure 3:** Evolution of suspected AFP cases in Niger from 1998-2021

Patient history and clinical presentation are presented in Tables II and III.

**Table II:** Distribution of patients based on contact with a suspected AFP case, onset of symptoms at the beginning of the illness, and their hospitalization during the period from 1998-2021

Variables	Sample size	Percentage (%)
**Case contact**		
yes	4 432	65,09
no	2 377	34,91
**Total**	**6 809**	**100**
**Pain**		
yes	1 777	61,04
no	1 134	38,96
**Total**	**2 911**	**100**
**Fever**		
yes	6 857	96,07
no	280	3,93
**Total**	**7 137**	**100**
**Hospitalization**		
yes	953	12,99
no	6 383	87,01
**Total**	**7 336**	**100**

**Table III:** Distribution of patients by mode of onset, location, asymmetry, progression of paralysis, and doses of OPV received in Niger from 1998 to 2021

Variables	Sample size	Percentage (%)
**Sudden onset of paralysis**		
yes	7 031	98,97
no	73	1,03
**Total**	**7 104**	**100**
**Location of paralysis (limb)**		
1	64	23,18
2	198	71,73
3	68	2,17
4	8	2,92
**Total**	**276**	**100**
**Asymmetry of a lower limb**		
yes	2 383	94,35
no	4 316	5,65
**Total**	**6 699**	**100**
**Number of OPV doses received**		
0	165	1,72
1-2	2 203	23,05
3-10	4 161	43,54
> 10	3 027	31,69
**Total**	9 556	100

Patients who had received an injection prior to disease onset represented 10.32% of the total. Paraclinical data are presented in Table IV.

**Table IV:** Distribution of patients based on the characteristics of collected stool samples (1998-2021)

Variables	Sample size	Percentage (%)
**Condition of stool samples**		
adequate	8 419	88,7
not adequate	1 073	11,3
**Total**	**9 492**	**100**
**Final culture**		
negative	6 587	70,26
suspected poliovirus	866	9,24
suspected poliovirus + non-polio enterovirus	36	0,38
non-polio enterovirus	1 886	20,12
**Total**	9 375	**100**
**Final classification of cases**		
spolio confirmed	58	0,80
compatible	169	2,35
not included	6 960	96,85
**Total**	**7 187**	**100**

The different delays in case notification, investigation and admission are summarized in Tables V and VI.

**Table V:** Distribution of patients by time from onset to notification, from notification to case investigation, and from onset to case admission in Niger (1998-2021)

Variables	Sample size	Percentage (%)
**Time from onset to notification (in days)**		
<1	374	5,2
2-3	1 209	16,83
>3	5 598	77,97
**Total**	**7 181**	**100**
**Time from notification to case investigation**		
≤ 48 hours	9 457	98,07
> 48 hours	186	1,93
**Total**	9 643	**100**
**Time from onset to admission (in days)**		
1	84	11,18
2-3	154	20,5
>3	513	68,32
**Total**	**751**	**100**

**Table VI:** Distribution of patients by time between two specimens, time from shipment to receipt at the laboratory, and time from onset to first specimen (1998-2021)

Variables	Sample size	Percentage (%)
**Time from first to second sampling**		
≤ 48 hours	7 248	99,72
> 48 hours	20	0,28
**Total**	**7 268**	**100**
**Time from sampling to laboratory shipment**		
≤ 72 hours	477	6,82
> 72 hours	6 517	93,18
**Total**	**6 994**	**100**
**Time from shipment to receipt at the laboratory**		
≤ 72 hours	7 124	77,66
> 72 hours	2 049	22,34
**Total**	**9 173**	**100**
**Time from onset to first sampling (in weeks)**		
2	6 326	87,37
4	570	7,87
6	238	3,28
> 6	106	1,48
**Total**	**7 240**	**100**

In 22% of cases, notification was made within 3 days or less after the onset of illness. In 2% of cases, the investigation took more than 48 hours. In 34% of the cases, the time between the onset of illness and admission was less than or equal to 3 days.

Table VII shows the different levels reached by the indicators according to the thresholds defined by the WHO.

**Table VII:** Breakdown of AFP surveillance system indicators in Niger, 1998-2021

Indicator	WHO standard	Niger results	Commentaire	Comment
Rate of non-polio AFP in children under 15 years of age	> 2	1,5	Beaucoup de districts étaient silencieux, ne notifiaient pas ou notifiaient moins, surtout au moment de la réorganisation du système sanitaire du Niger à partir de 2014 (passage de 44 à 72 districts).	Many districts were silent, did not report, or reported less, especially at the time of the reorganization of the health system in Niger from 2014 (increase from 44 to 72 districts).
Percentage of AFP cases for which 2 samples are collected within 14 days	≥ 80 %	100 %	L'objectif est atteint du fait que l'investigation se fait en collaboration avec les agents du district et les partenaires en plus des agents du centre de santé intégré.	The objective was achieved because the survey was carried out in collaboration with district agents and partners, in addition to the agents of the integrated health center.
Percentage of AFP cases with 60-day follow-up	≥ 80 %	92 %	Objectif atteint, voire dépassé, grâce à l'implication des acteurs.	The goal was achieved and exceeded thanks to the involvement of all stakeholders.
Percentage of stool samples arriving at the laboratory in good condition	≥ 80 %	89 %	La formation des agents et la mise en place d'un système de surveillance cas par cas pour la poliomyélite au niveau national à travers la DSRE.	Training of agents and implementation of a case-based surveillance system for poliomyelitis at the national level through the DSRE.
Percentage of samples for which results are sent within 28 days of receipt in the laboratory	≥ 80 %	83 %	Un mécanisme de transport était mis en place avec la sous-traitance des compagnies de transport.	A transport mechanism was set up by subcontracting transport companies.
Percentage of samples for which a nonpoliovirus enterovirus is isolated	> 10 %	30 %	Le renforcement de capacité des laboratoires et leur implication dans le système de surveillance surtout avec des modules spécifiques dans le document du système intégré des maladies et riposte (SIMR) plus précisément la SIMR 4.	Capacity building for laboratories and their involvement in the surveillance system, especially with specific modules in the Integrated Disease and Response System (IDRS) document, particularly IDRS 4.
Percentage of cases investigated within 48 hours of notification	≥ 80 %	98 %	L'objectif est aussi atteint du fait de la mise en place d'un système de surveillance à base communautaire avec l'implication des relais communautaires. Plusieurs stratégies déterminantes, dont celle de l’OMS dénommée autovisual AFP detection and reporting (AVADAR), qui permet de déclencher une alerte au niveau communautaire. Cette alerte passe par le centre de santé intégré, le district sanitaire, le DRSP, la DSRE, l’OMS pays et jusqu'au niveau OMS sous régional et dès là un rapport d'investigation est attendu au niveau périphérique dans les 24 à 48 heures qui suivent.	This goal has also been achieved through the establishment of a community-based surveillance system involving community relays. There are several key strategies, including the WHO's Automatic Visual AFP Detection and Reporting (AVADAR) strategy, which triggers an alert at the community level. This alert goes through the integrated health center, the health district, the DRSP, the DSRE, the WHO country, and up to the WHO subregional level. An investigation report is then expected at peripheral level within 24 to 48 hours.

## Discussion

Several indicators met and some exceeded WHO thresholds. This is the result of several efforts and strategies implemented, from the vaccine response to the community surveillance system set up at the country level with the support of partners, including WHO. The only indicator that remains below the WHO threshold is the rate of non-polio AFP in children under 15 (1.5 instead of > 2). In 2014, Niger's health system underwent a reorganization with the creation of several districts from existing ones, increasing their number from 44 to 72, with a variable adaptation period. Resources and infrastructure (especially cold chains, vaccine reagents and consumables) have not always kept pace. Only the annual action plans of the old health districts have been prepared, validated and funded. The activities of the new districts are based on the strict implementation of those of the old districts, where they are still considered integrated health centers of the parent districts. Monitoring indicators are expected from the new districts in the same way as from the old ones. Due to lack of resources, the new districts did not report suspected AFP cases. This failure to include all data largely explains the differences in numbers between indicators. Case reporting at the community level was done through weekly telegrams on notifiable diseases, without systematic investigations. These deficiencies persisted for several years at the beginning of surveillance. This and other studies show a sex-ratio in favor of males [1, 6, 7, 18]. Boys, who are more often left to their own devices in early childhood than girls, are exposed to sources of infection (viral, parasitic, bacterial, etc.) at an earlier age through play and exposure to unhealthy environments. AFP is more prevalent in children under 5 years of age [2, 4, 10]. Viruses are common in the environment. Other causes, such as multiple intramuscular injections during anti-infective treatments, can lead to debilitating sequelae. In addition, health care workers are more likely to seek out and report suspected cases of AFP in children under 5 years of age than in older children. Children under the age of 5 are more accessible because they are covered by most vertical programs for immunization, drug distribution, and malnutrition control through the various nutrition recovery centers. In most patients, AFP is associated with fever, which distinguishes it from Guillain-Barré syndrome. Fever is present before, during, and after the onset of paralysis in neuritis. It is rare in transverse myelitis. Fever may also be associated with one or more other infectious diseases. When associated with flaccid paralysis that develops rapidly within 72 hours and resolves soon after the onset of paralysis, it is suggestive of poliomyelitis [8].

In this series, nearly one-third of AFP patients presented with asymmetric limb paralysis, most commonly affecting the lower limbs and proximal muscles (quadriceps, deltoid) in a relatively anarchic fashion, which is characteristic of poliomyelitis [3, 5, 8]. The intensification of vaccination campaigns in recent years in Niger may explain the decline in polio cases. In addition, surveillance is being strengthened through new strategies such as Auto-Visual AFP Detection and Reporting (AVADAR). This is a SMS-based mobile software application designed to improve the quality and sensitivity of AFP surveillance by health workers and key informants in health facilities and local communities. Finally, environmental surveillance has been improved in Niger since 2014 through sentinel sites, which were then scaled up between 2015 and 2018.

The quality and timeliness of stool samples – ideally less than 72 hours after exposure – is essential for successful diagnosis [18, 19]. It is not always possible to attribute certain AFP cases to non-polio enteroviruses. The incidence of wild and vaccine polioviruses and non-polio enteroviruses, particularly in asymptomatic children, varies by diagnostic context [2, 18]. The reversion to neurovirulence of vaccine poliovirus could be explained by genetic variability during which these strains acquire a partially neurovirulent phenotype in the gut. Such post-vaccination paralysis should be systematically studied as long as oral poliovirus vaccine remains in use. In some countries, such as France, the injectable vaccine is preferred because of these reversions [12, 14, 17]. According to WHO, at least 80% of cases of acute flaccid paralysis must be accompanied by two stool samples taken between 24 and 48 hours apart and within the following two weeks [14]. A surveillance system is considered effective if 80% of samples are collected within 14 days [14]. In Niger, stool samples are tested at the Institut Pasteur reference laboratory in Dakar. The last case of wild poliovirus was reported in 2012 in the Keita health district (Tahoua region), and the country was certified wild poliovirus-free by the Regional Certification Commission for Polio Eradication in Africa in July 2016. However, since September 2020, cases of vaccine-derived type 2 poliovirus have been observed, with 9 cases imported from Nigeria, Togo, and Côte d’Ivoire [13, 16].

From 2020, the involvement of health workers in the management of the COVID-19 pandemic and the closure of air and land routes had a negative impact on AFP surveillance. This resulted in a decrease in the number of reported cases, poor quality specimens and delays in their arrival at regional reference laboratories: 365 samples remained in storage at the central level for several months. It is therefore necessary to equip the country with a specialized laboratory to increase the speed of poliovirus detection to distinguish between wild and vaccine-derived polioviruses. Surveillance must be maintained, with emphasis on: a) systematic collection of all suspected AFP cases; b) submission of suspected stool samples to the reference laboratory, following the conditions for collection, storage and submission; c) systematic investigation of all AFP cases; and d) initiation of response activities.

Since 1998, Niger's surveillance system has performed well, with most indicators at or above 80%, in line with WHO recommendations (Table VII). In this series of studies, the highest prevalence of polio cases was observed in the Maradi region. The year 2018 had the highest number of cases. The vaccination status of the children showed an acceptable level of immunity against polio. Disease progression over 3 days was more common in unvaccinated patients. New WHO recommendations on vaccines and AFP surveillance are essential for polio eradication. In Niger, continued epidemiologic and laboratory surveillance is essential to maintain certification as polio-free at a critical time in the global eradication effort. However, this can only be achieved through continued clinical surveillance for AFP, including appropriate reporting and testing protocols.

## Conclusion

Over the past 24 years, active surveillance of AFP in Niger has been generally acceptable according to the performance indicators. However, there is still work to be done both at population level (compliance with food and environmental hygiene measures) and at health service level (community involvement in all surveillance activities through education and information). Epidemiological surveillance is important to maintain certification as a polio-free country. This can be achieved through continued vigilance regarding AFP.

## Study funding

The study did not receive any funding.

## Authors’ contributions

ABDOULAYE Z and ALKASSOUM S.I: Epidemiologists and collaborators in the public health department of Abdou Moumouni University, involved in the research from protocol to data analysis.

MARIE M.M: 8th year medical student, involved in data extraction

GARBA AA, HAROUNA M, ABDOL KADIR I.M, MAHAMANE D.M: internists and infectiologists in the regions of Dosso, Maradi, Zinder and UAS, involved in data collection and analysis.

ADEHOSSI E.O: Full Professor of Internal Medicine, involved in accessing certain sources of information and writing the article.

AKA J: Associate Professor in Epidemiology, for appreciation of all the work.

SAIDOU M: Professor of Bacteriology and Virology, for validation of all the work.

## Declaration of interest

The authors declare no conflict of interest.
